# Editorial: Application of noninvasive neuromodulation in cognitive rehabilitation

**DOI:** 10.3389/fneur.2023.1333474

**Published:** 2023-12-07

**Authors:** Pengxu Wei, Le Li, Giuseppe Lanza, Mariagiovanna Cantone, Ping Gu

**Affiliations:** ^1^Alzheimer's Disease and Cognitive Rehabilitation Committee, Chinese Association of Rehabilitative Medicine, Beijing, China; ^2^Institute of Medical Research, Northwestern Polytechnical University, Xi'an, China; ^3^Department of Surgery and Medical-Surgical Specialties, University of Catania, Catania, Italy; ^4^Clinical Neurophysiology Research Unit, Oasi Research Institute-IRCCS, Troina, Italy; ^5^Neurology Unit, Policlinico University Hospital “G. Rodolico-San Marco”, Catania, Italy; ^6^Department of Neurology, The First Hospital of Hebei Medical University, Shijiazhuang, Hebei, China

**Keywords:** cognitive impairment, cognitive training, rehabilitation, noninvasive neuromodulation, neurological diseases

Alzheimer's disease (AD), Parkinson's disease (PD), cerebrovascular diseases, and many other neurological diseases are common causes of cognitive dysfunction. Mild cognitive impairment (MCI) may progress into dementia, which imposes a severe burden upon patients and their caregivers. Currently, given the lack of disease-modifying treatments, this Research Topic aimed at widening the knowledge on cognitive training and noninvasive neuromodulation therapies including transcranial magnetic stimulation (TMS), transcranial alternating current stimulation (tACS), and focused ultrasound-mediated blood-brain barrier (BBB) opening, which may improve cognitive function and/or reduce deposition of pathologic substances, such as amyloid β (Aβ) protein.

Sargénius et al. found that for children with acquired brain injury, a 8-week cognitive rehabilitation program resulted in a sustained improvement of executive function lasting for two years. This finding indicates that the 8-weeks training might shape some factors, which facilitated the improvement of cognition in a 2-year period. Similarly, a 4-week video game training in the elderly enhanced cognitive abilities and the training-induced boost in sustained attention and preservation of cognitive improvement could be observed 6 months later (1). Also, a short duration (ten 60–75 min sessions over 5–6 weeks + four 75-min sessions at 11 and 35 months) of specific types of cognitive training for the elderly led to improved cognitive abilities, even at 10 years after intervention (2). These long-term cognitive protection effects suggest that a short-term cognitive training may be a cost-effect intervention for treating or preventing cognitive impairments, although the reason why a short-period training resulted in such long-lasting effects is not fully understood. Additionally, more studies on the long-term effects of cognitive intervention are expected.

TMS or tACS can transiently modulate brain activity and improve cognitive function. Chen et al. found that high-definition theta tACS resulted in weakened connections between some brain regions during both resting state and a memory task in health subjects. Yan et al. performed a systematic review and meta-analysis, which demonstrated the effects of TMS on cognitive improvement in patients with MCI and AD. The applied stimulation sites (i.e., the dorsolateral prefrontal cortex and the cerebellum), parameters (e.g., frequency of 5 Hz and 10 Hz), and protocols (such as the intermittent theta-burst stimulation) influenced the effects of TMS interventions.

Low-intensity ultrasound (LIUS) beams oscillate intravenous microbubbles to generate acoustic cavitation that, transiently and reversibly, is able to open tight junctions in capillaries and the BBB in the superficial dorsolateral prefrontal cortex as well as deeply located hippocampus and entorhinal cortex (3). This method can be used also for targeted noninvasive delivery of genetic vectors and cells. Zhou et al. reviewed the effects of LIUS combined with microbubble-mediated BBB opening on reducing Aβ and tau pathologies, stimulating neurogenesis, and improving cognitive performance in both AD mice and patients with and without delivering any drugs. Interestingly, LIUS combined with intravenous microbubbles resulted in reduction in amyloid burden and cognitive improvement in PD-related dementia (4), led to decreased amyloid accumulation, and slowed down the reduction of glucose metabolism in mild AD patients (5).

Taken together, the improvement in cognitive function found in the above studies may stem from a variety of factors, including reduction in amyloid burden, improvement of glucose metabolism status, increases of the activity of local brain regions, and strengthening of functional connectivity between brain areas.

Concluding, cognitive training and noninvasive neuromodulation therapies are both safe and effective when rationally applied and in selected cases (6). One of possible applications is to use these methods to decelerate the exacerbation of cognitive impairment (e.g., due to AD) or to improve cognitive function in patients with secondary dementia, such as vascular-related cognitive disorders (7); the other main application can be the disease prevention and differential diagnosis with other, non-degenerative, conditions (8) (more evidence is needed to substantiate these possible applications). For instance, pathologic changes and cognitive decline of AD occur continuously over a long period. Based on different genetic backgrounds, the burden of Aβ/tau pathology, neurodegeneration, and cognitive symptoms, three variants of AD are proposed, namely the autosomal dominant AD, the apolipoprotein-E (APOE) ε4-related sporadic AD, and the APOE ε4-unrelated sporadic AD. What these three variants have in common is that the brain pathological process may occur at the age of around 20, i.e., several decades before the typical dementia onset (9). These low-cost, safe, and effective strategies can be applied during such a long period to enhance brain resilience (e.g., via cognitive training) that may prevent or delay cognitive impairment due to AD and/or to reduce deposition of pathologic substances (e.g., the Aβ protein), eventually modifying the progression of AD. Moreover, the parameters of interest of each therapy can be optimized to improve efficacy and feasibility. Additionally, the application of these approaches as a combined therapy is also worth of investigation, considering that they may improve cognitive function through a variety of mechanisms. For instance, LIUS combined with microbubble-mediated BBB opening is applied to reduce Aβ pathology in its very early stage, TMS and/or tACS are applied to modulate brain connectivity, and cognitive training is used to increase the so-called “cognitive reserve” for subjects at the early stage of AD pathology (MCI or even earlier).

Future studies on larger samples are needed, based on careful sample selection, rigorous methodology, and follow-up. In the meantime, the high-quality translational studies included in this Research Topic may help to disentangle the multifaceted aspects of this complex scenario.

## Author contributions

PW: Conceptualization, Writing – original draft. LL: Conceptualization, Writing – original draft. GL: Conceptualization, Writing – original draft. MC: Conceptualization, Writing – original draft. PG: Conceptualization, Writing – original draft.
